# ID 125 - Revisão Sistemática e Metanálise da Efetividade e da Segurança do Sistema Flash de Monitorização da Glicose por Escaneamento Intermitente em Indivíduos com Diabetes Mellitus

**DOI:** 10.5327/2237-9622.2025.v34s1.125

**Published:** 2025-11-25

**Authors:** Vania dos Santos Nunes Nogueira, Mayra Souza Botelho, Mariana Andrades Fiorini Monteiro Novo, Julia Simões Corrêa Galendi, Vania dos Santos Nunes Nogueira

**Keywords:** diabetes mellitus, variabilidade glicêmica, sistema flash de monitorização intermitente da glicose, hipoglicemia, revisão sistemática

## Abstract

**Introdução:**

O diabetes mellitus (DM) pode levar a complicações graves, como doenças cardiovasculares, insuficiência renal e cegueira. Além da hiperglicemia, a variabilidade glicêmica tem sido considerada como um fator de risco para o surgimento destas complicações. Por conta disso, o tratamento do DM deve abordar a hiperglicemia crônica, hipoglicemia e variabilidade glicêmica. Como métodos para monitorização contínua da glicemia têm-se: a automonitorização da glicemia capilar (AMGC); os sistemas de monitoramento contínuo de glicose (SMCG), e mais recentemente o sistema flash de monitorização da glicose por escaneamento intermitente (SFMG). O objetivo deste trabalho foi avaliar por meio de ensaios clínicos randomizados (ECR) a eficácia do SFGM comparado à AMGC no tratamento de indivíduos com DM1 ou DM2 em insulinoterapia.

**Método:**

Foi realizada uma revisão sistemática de acordo com as diretrizes metodológicas do Ministério da Saúde de revisões sistemáticas de ECR (registro PROSPERO: CRD42024562805). Foram incluídos ECR, nos quais os pacientes com DM e em uso de insulina foram alocados ao SFMG ou à AMGC. Os desfechos analisados foram HbA1c (%), tempo em hipoglicemia (glicemias abaixo de 70mg/dl), satisfação do paciente no tratamento do DM [Diabetes Treatment Satisfaction Questionnaire (DTSQ)], evento adverso e tempo no alvo (glicose entre 70 a 180 mg/dl). Embase, MEDLINE e CENTRAL foram as bases eletrônicas pesquisadas. O processo de seleção, avaliação do risco de viés (ferramenta RoB 2) e extração dos dados foram realizados em pares e independentemente. Como estimativa de efeito da intervenção foram calculados o risco relativo (RR) e a diferença de média (DM), ambos com o intervalo de confiança de 95% (IC 95%). Foram realizadas metanálises (modelo de efeitos aleatórios) usando o software estatístico Stata 18. A qualidade da evidência foi gerada de acordo com as Diretrizes Metodológicas do Ministério da Saúde do Sistema GRADE.

**Resultados:**

Foram identificadas 1.171 referências, tendo sido incluídos 17 RCT. A metanálise da HbA1c aferida na última consulta de seguimento favoreceu a intervenção (DM: -0,25%, IC 95%: -0,39 a -0,10%, moderada qualidade da evidência). Em relação à satisfação do paciente, a metanálise também favoreceu a intervenção (DM: 4,5, IC 95%: 2,28 a 6,82, moderada qualidade da evidência). No que se refere ao tempo em hipoglicemia, a metanálise também favoreceu a intervenção, porém a qualidade da evidência foi baixa (DM: -0,14%, IC 95%: -0,21 a -0,06%). Em relação ao tempo no alvo, a metanálise não evidenciou diferença entre os grupos, mas a qualidade da evidência foi muito baixa (DM: 0,02%, IC 95%: -0,05 a 0,1%). Os eventos adversos foram mais frequentes no SFMG (RR: 3.32, IC 95%: 1.79 a 6.14, moderada qualidade da evidência).

**Conclusão:**

Em indivíduos com DM e uso diário de múltiplas doses de insulina, o SFMG comparado à AMGC resultou em diminuição da HbA1c e melhora na satisfação do paciente no tratamento do DM; moderada qualidade da evidência. A intervenção favoreceu também o tempo em hipoglicemia, e não mostrou diferença de tempo da glicose no alvo, porém a qualidade da evidência foi baixa e muito baixa, respectivamente. A frequência de eventos adversos foi maior no grupo intervenção, porém foram eventos cutâneos não considerados graves.

